# A Hospital Saturday Fund in Danger

**Published:** 1896-09-19

**Authors:** 


### The Hospital

An Institutional Journal of

The Medical Sciences and Hospital Administration, with

AN EXTRA NURSING SUPPLEMENT.

Vol. XX.—No. 521.] [Sept. 19, 1896.

### A Hospital Saturday Fund in Danger.

Bricks and mortar have proved the ruin of many a good scheme, and it is with a disquietude which we feel sure must be shared by many well wishers of the Birmingham Hospital Saturday movement, that we hear of a proposal to make use of the machinery, if not actually to utilise the funds, of that useful and thriving organisation, for the furtherance of a building project which is of a purely speculative nature. While having within it many elements of success—a success which, however, would in no way help the Hospital Saturday Fund—this project has also in it many elements of failure which, if it were to occur, would involve the Fund in grave disaster; and it must not be forgotten that, whether success or failure be its portion, it is a scheme having nothing whatever to do with the prime object of the Hospital Saturday movement.

The scheme as at present put forward seems to be somewhat inchoate, and involves the enlistment of the Birmingham working man in the promotion of many objects in regard to which the working men of England are at the present moment far from being of one opinion. While we are told that the new scheme is to be absolutely independent of the trades unions, and "will embrace non-society as well as society men, and trades who have no societies," it is to be noticed that one of its first aims is "to endeavour, as far as lies in the power of the members, to find occupation for those residing in the division who are out of work." Is this true? Is there the slightest likelihood that when the horizon is dark with trade disputes the society men will throw aside all their trade teaching and all their trade traditions to "find occupation" for those non-society men who are out of work? Why, the very existence of the unions depends on their success in preventing the out-of-works, who always hang around every trade, from accepting the work offered to them when trade disputes are in progress. We have nothing to do here with such matters, but we know well enough the feelings of working men, the extraordinary, and in a sense the laudable, clannishness of men who, for the sake of what we may term trade honour, are ready to suffer the extremest privations, and for the sake of the good of the "union," will, at the nod of the "secretary," throw up good wages and prosperity and cast themselves into the ranks of the unemployed; and the more we know of the wonderful discipline to which working men, and often the cream of working men, are willing to subject themselves for the sake of furthering the success of their trade organisations, the more speculative seems an adventure which trusts for its success to the lion and the lamb lying down together, and thinks that society and non-society men will combine in times of trouble to find work for the unemployed! Yet on this hope and this speculation is to hinge the success of a project to which the credit, perhaps even the existence, of that hitherto great philanthropic movement, the Birmingham Hospital Saturday Fund, is to be pledged.

The great truth which the people of Birmingham have to recognise, and on the recognition of which the permanence of their Hospital Saturday Fund depends, is that hospitals are charitable institutions. To their honour be it said that the working people of Birmingham contributed their weekly pence, just as did those who subscribed their guineas or their scores of guineas, as a charity to their fellow men. Truly the gift fell mostly to people of their own class in life, but that could not be helped; the men who gave gave freely, without thought of receiving ought in return. This was good, and the working men of Birmingham were an example of charity to others. But in regard to this new proposal we are told that there is "no philanthropy in it." What claim has it, then, on the support of the Hospital Saturday Fund, which is rooted in philanthropy?

One of the great dangers to which working-class organisations are always exposed is that of being captured by cliques and faddists for their own purposes; and the more hopeful and enthusiastic the leaders, the greater is the danger. We all know the contagious enthusiasm of Mr. Smedley, and the power which he is able to exercise over his fellow-workers; but it should be impressed on those who have any say in the matter that, once leave on one side the great object for which the Birmingham Hospital Saturday Fund was started, namely, the assistance of the hospitals—once admit that the organisation may be used for other purposes—and it is open to anybody to manipulate it for the advancement of any project in which he may be interested.

It would be a pity to see an organisation which was founded upon, and has always been held up as proof of the charity of the Birmingham working men, made use of, however indirectly, for the furtherance of commercial speculations.

